# Editorial: The role of genetics and non-coding RNAs in atrial fibrillation

**DOI:** 10.3389/fcvm.2022.997700

**Published:** 2022-08-03

**Authors:** Valeria Novelli, Fadi G. Akar

**Affiliations:** ^1^Unit of Immunology and Functional Genomics, Centro Cardiologico Monzino, IRCCS, Milan, Italy; ^2^Department of Internal Medicine, Section of Cardiovascular Medicine, Yale School of Medicine, New Haven, CT, United States; ^3^Department of Biomedical Engineering, Yale University Schools of Engineering and Applied Sciences, New Haven, CT, United States

**Keywords:** atrial fibrillation, genetics, non-coding RNAs, LMNA, genetic factor

Atrial fibrillation (AF) is a global public health epidemic that impairs quality of life and increases the risk for stroke, dementia, heart failure, and sudden cardiac death. AF is the most prevalent arrhythmic disorder, affecting over 33 million individuals worldwide. The highly prevalent nature of AF stems from the fact that multiple very common risk factors promote its incidence, including but not limited to ischemic heart disease, heart failure, alcohol consumption, valvular heart disease, and hypertension. All of these factors are modulated not only by environmental factors but also by genetic predisposition. In fact, an increasing body of evidence implicates genetics *per se* in the early development of lone AF independently of the aforementioned traditional risk factors. In this Research Topic, we introduce a series of articles that focus on the role of common and rare genetic variants as well as non-coding RNAs in the pathogenesis of adverse atrial remodeling and AF.

In the article by Chen X. et al., the authors performed a systematic meta-analysis across 7 different studies to clarify the putative role of a single nucleotide polymorphism (SNP), rs13216675 T>C, in AF risk. They concluded that this variant near *GJA1*, encoding the gap junction protein, Cx43, is significantly associated with an increased propensity for AF (Chen X. et al.). Those findings highlight the need for further studies to elucidate underlying mechanisms including gene–environmental interactions for the prevention or management of AF.

Shifting from a polygenic architecture of AF to a monogenic component, Pessente et al. investigated the presence of *LMNA* variants as a putative cause of “lone AF.” The authors analyzed a cohort of 101 probands with a clinical diagnosis of lone AF, identifying a total of eight individuals, three probands, and five first-degree relatives, carrying three different *LMNA* missense variants (Pessente et al.). They provided evidence that patients carrying rare *LMNA* variants appear to be at higher risk of arrhythmic events. Their findings reinforced the premise that the initial presentation of so-called laminopathies may indeed be in the form of lone AF.

In another original paper, Zhu et al. explored the relationship between circular RNAs (circRNA) and associated miRNA-mRNA regulatory networks in the pathogenesis of human AF in the setting of valvular heart disease, a major risk factor for AF. They methodically identified differentially expressed circRNAs in left atrial appendages of patients with or without persistent AF (Zhu et al.). Gene Ontology (GO) followed by KEGG pathway analysis revealed the importance of circRNA-mediated dysregulation of cAMP and Wnt signaling in the development of AF in patients with valvular heart disease.

A major risk factor in the progression of AF from paroxysmal to chronic forms is the development of adverse structural remodeling, in general, and atrial fibrosis, in particular. In a rat model of chronic angiotensin II infusion, Xiao et al. demonstrated effective suppression of atrial fibroblast proliferation and migration by miR-205, an effect that was apparently mediated *via* inhibition of JNK signaling. These intriguing findings highlighted a miR-205 regulated molecular signaling axis in adverse atrial structural remodeling that warrants further investigation.

Finally, the role of metabolic disease in AF is very well established. Specifically, obesity is now considered a major independent risk factor for AF, likely by promoting inflammation and oxidative stress (1). In this Research Topic, Chen S. et al. extended the mechanistic link between obesity and AF by providing a common genetic basis for both epidemics. Specifically, they found what appears to be a causal association between genetic factors that promote elevated birth weight and vulnerability to AF later in life.

Collectively, the studies presented in this Research Topic highlight the need for accelerating the search for genetic factors that influence the pathogenesis of AF, both directly and indirectly by promoting its established risk factors. Such studies are likely to reveal new targeted strategies for treatment and/or prevention of this major public health epidemic.

## Author contributions

All authors listed have made a substantial, direct, and intellectual contribution to the work and approved it for publication.

## Funding

FGA is supported by grants from the National Institutes of Health 1R01HL149344 and 1R01HL148008.

## Conflict of interest

The authors declare that the research was conducted in the absence of any commercial or financial relationships that could be construed as a potential conflict of interest.

## Publisher's note

All claims expressed in this article are solely those of the authors and do not necessarily represent those of their affiliated organizations, or those of the publisher, the editors and the reviewers. Any product that may be evaluated in this article, or claim that may be made by its manufacturer, is not guaranteed or endorsed by the publisher.
